# Supplementary material for: Rational design of spontaneous reactions for protecting porous lithium electrodes in lithium–sulfur batteries
Source: Nat Commun. 2019 Jul 19;10:3249. doi: 10.1038/s41467-019-11168-y (PMC6642196; doi:10.1038/s41467-019-11168-y)
Supplement: Supplementary file 1 — Supplementary Information [file 41467_2019_11168_MOESM1_ESM.pdf]

**Supplementary information**

# Rational design of spontaneous reactions for protecting porous Li electrodes in lithium-sulfur batteries

Ren et al.

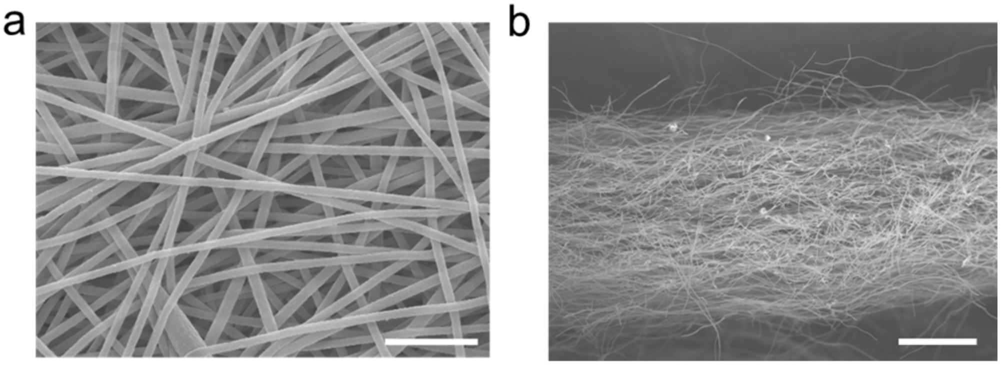


**Supplementary Figure 1 Morphology of the pristine CNF.** (a) SEM of the surface of the pristine CNF; (b) cross-sectional SEM of the pristine CNF matrix. Scale bar for a and b is 5 μm and 20 μm, respectively.

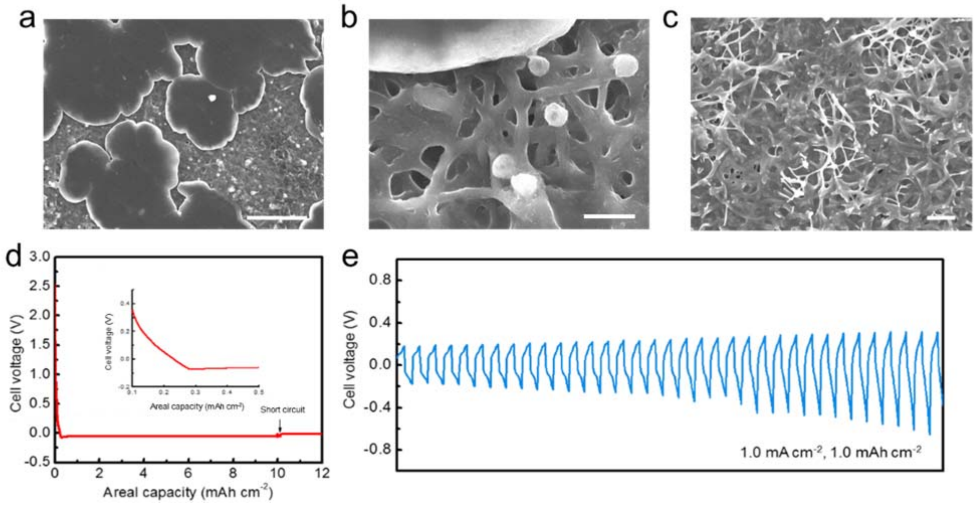


**Supplementary Figure 2 Li plating characteristics of pristine CNF.** (a, b) SEMs of the top surface (separator side) of the CNF after short circuit (induced by Li plating) at different magnifications. (c) SEM image of the top surface (current collector side) of the CNF after Li plating. (d) The voltage profile of Li/CNF half cell, which was discharged at 0.5 mA cm$^{-2}$ until the cell reached short circuit. (e) The voltage profiles of the symmetric cell assembled with the CNF electrode plated with an equivalent amount Li (8.0 mAh cm$^{-2}$). Under the same testing condition for the symmetric cell, the CNF electrode with electrodeposited Li showed an even larger overpotential compared with the bare Li metal (Fig. 5d). Scale bar for a-c is 50 µm, 5 µm and 10 µm, respectively.

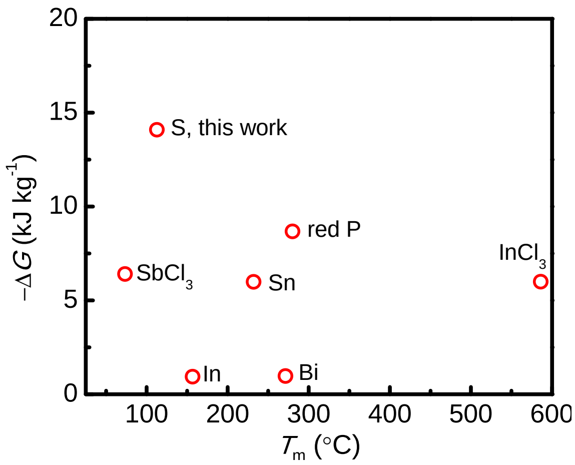


**Supplementary Figure 3 Comparison of the Gibbs free energy of the lithiation reactions (vacuum, *T* = 0 K) and melting points of various materials.** The calculation of Gibbs free energy was based on the data from https://materialsproject.org/[1].

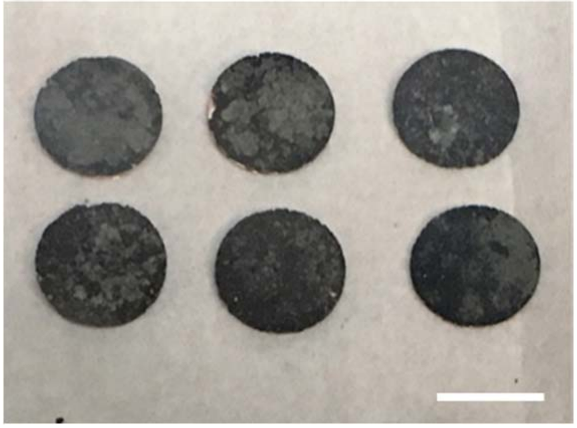

**Supplementary Figure 4 The digital photo showing the formed Li/S-CNF electrodes after mechanical pressing, with an variance in thickness of 3 µm.** Scale bar is 1 cm.

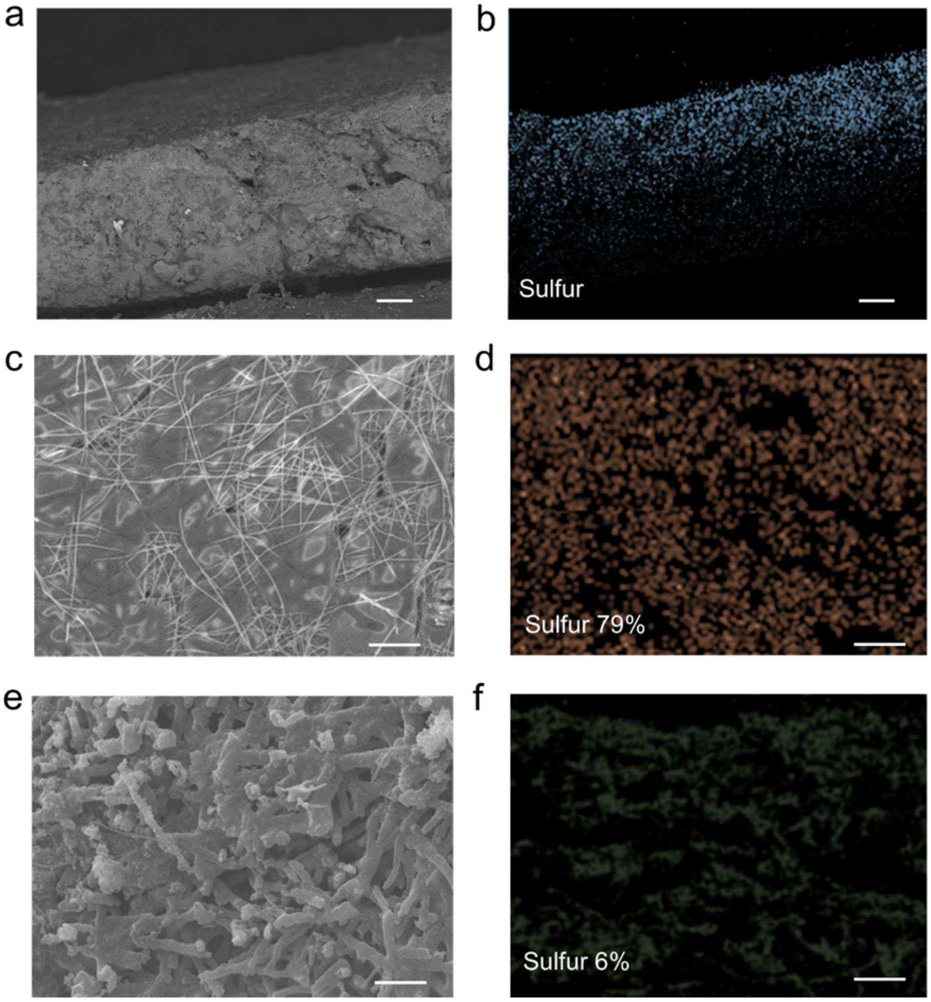


**Supplementary Figure 5 Characterizations of Li/S-CNF.** (a, b) Cross-sectional SEM of the Li/S-CNF (a), and its EDX (b); (c, d) SEM of the so-called $Li_2S$-rich surface of Li/S-CNF (c), and its EDX (d); (e, f) SEM of the so-called Li-rich surface of Li/S-CNF (e), and its EDX (f). The atomic fraction of sulfur was calculated considering sulfur, carbon and oxygen. Scale bar for a, b is 20 μm; scale bar for c-f is 10 μm.

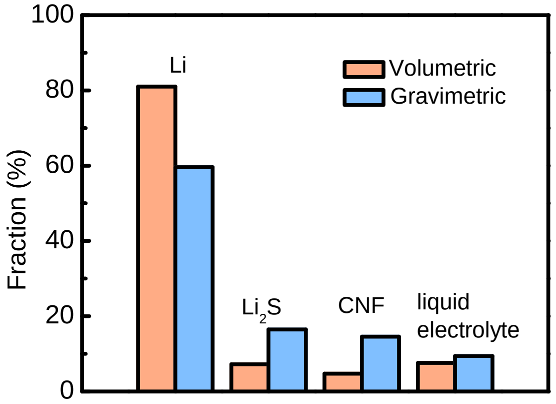


**Supplementary Figure 6 The calculation of gravimetric/volumetric fractions of Li/S-CNF electrode.**

( $\rho_{\mathrm{Li}}$ = 0.534 gcm$^{-3}$, $\rho_{\mathrm{Li_2S}}$=1.66 g cm$^{-3}$ , $\rho_{\mathrm{CNF}}$=2.25 g cm$^{-3}$, $\rho_{\mathrm{electrolyte}}$≈0.90 g cm$^{-3}$ ).

The Li-S/CNF electrode was compressed before characterization and testing. Before compression, the electrode is more porous and allows for the solution deposition to occur uniformly through it.

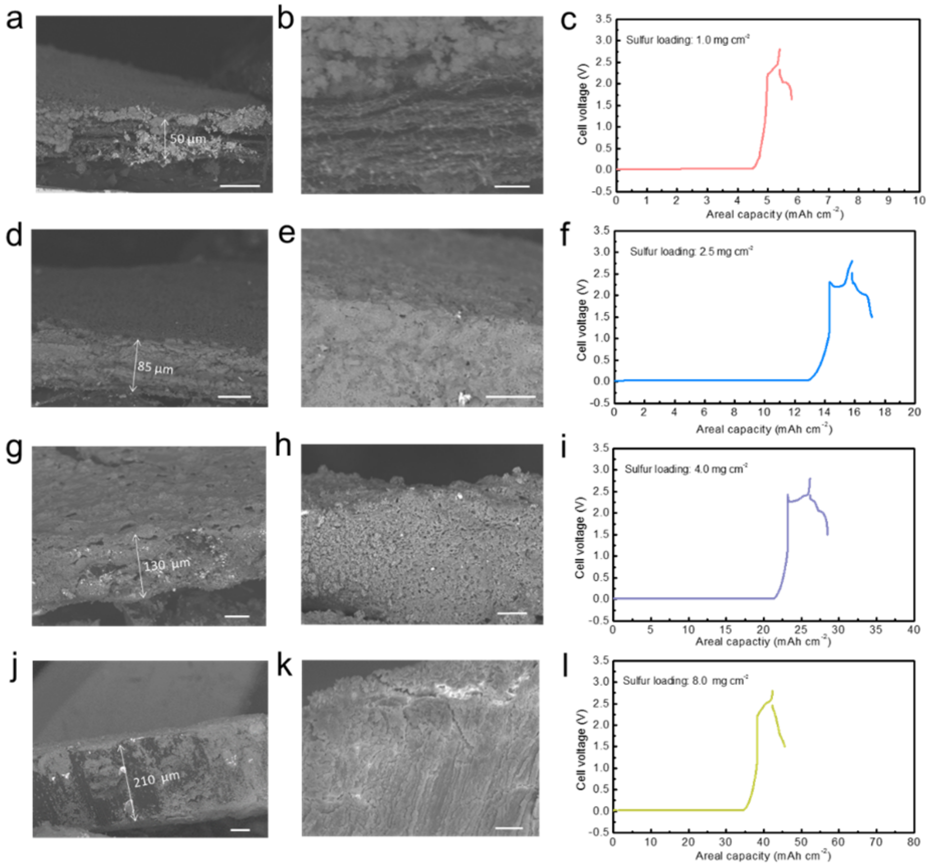


**Supplementary Figure 7 Cross-sectional SEMs of Li-S/CNF electrodes fabricated with different initial sulfur loadings and their delithiation (0.5 mA cm$^{-2}$) voltage profiles.** Sulfur loading: (a-c) 1.0 mg cm$^{-2}$; (d-f) 2.5 mg cm$^{-2}$; (g-i) 4.0 mg cm$^{-2}$; (j-l) 8.0 mg cm$^{-2}$. When charging up to 2.1 V, we can further observe the voltage profiles representing the delithiation of $Li_2S$. Scale bar for a, d, g, j is 50 µm; scale bar for b, e, h, k is 10 µm.

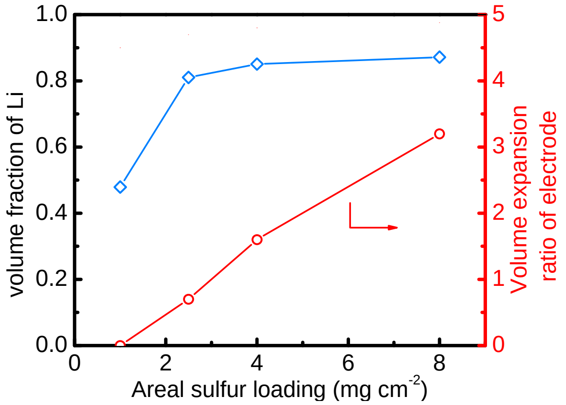


**Supplementary Figure 8 The impacts of the initial sulfur loading on the volume fraction of Li in the electrode and the volume expansion ratio of the electrode.**

**Supplementary Note 1**

The initial loading of sulfur can have a significant impact on the structural property of the Li electrode. At a higher sulfur loading, the exothermic reaction with Li can provide more heat to reduce the viscosity of molten Li and drive the wetting of Li on the carbon surface. From the cross-sectional SEM in **Supplementary Figure 7**, we can observe a higher initial sulfur loading results in a denser filling of the CNF matrix and a larger volumetric expansion. We further estimated the amount of Li infused inside the matrix using a half-cell setup with a large MFC-Li (16 mm in a diameter) as the counter electrode and a small Li/S-CNF (5 mm in diameter) as the working electrode. The corresponding voltage profiles are shown in **Supplementary Figure 7**c, f, i, l. Assuming complete Li stripping, the volume fraction taken up by Li can be derived. Herein, we specifically chose an initial sulfur loading of 2.5 mg cm$^{-2}$, which was almost the minimum loading value to trigger the uniform Li filling (**Supplementary Figure 8**). Besides, under this condition, the volume expansion ratio of the anode can be also maintained at a reasonable value (~70%).

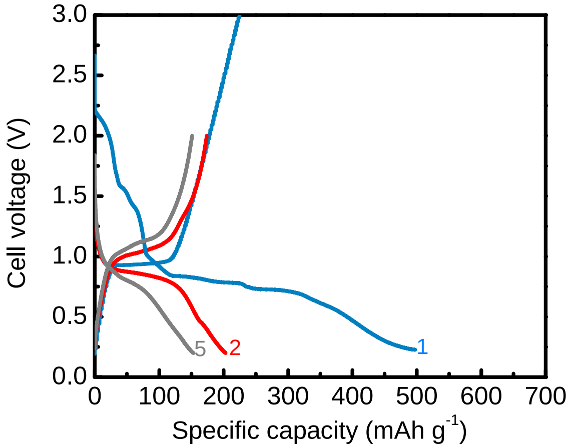


**Supplementary Figure 9 Discharge/charge voltage profiles for the half cell assembled with $BiF_3$ (604.5 mAh $g^{-1}$) coated Cu foil and a Li metal anode (16 mm in diameter).**

**Supplementary Note 2**

To fabricate the $BiF_3$/Cu electrode, the NMP dispersion of metal fluoride (60 mg $mL^{-1}$, with 10 mg $mL^{-1}$ PVDF binder) was uniformly drop casted to derive an areal loading of metal fluoride about 1.5 mg $cm^{-2}$ on the Cu foil (12 mm in diameter) and dried at 120 $^{o}$C to completely evaporate the solvent before assembly. The rate was set as 0.1 C based on the $BiF_3$ weight. The conversion from $BiF_3$ to Bi and LiF is irreversible in the ether based electrolyte, but Bi can be alloyed/dealloyed.

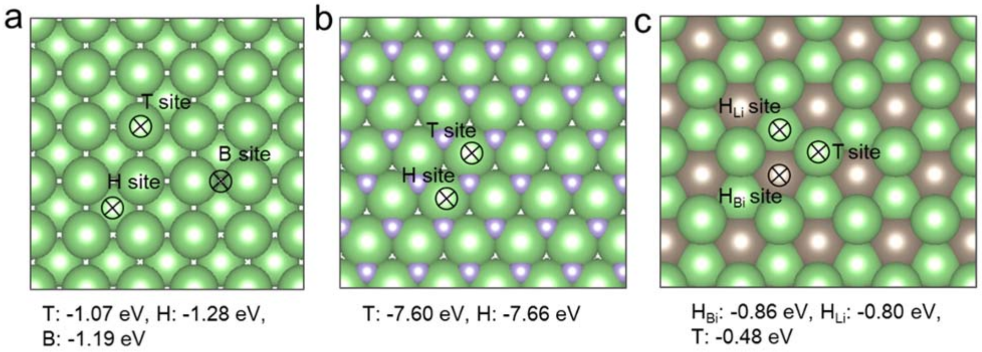


**Supplementary Figure 10 Adsorption sites and energies for Li adatom on different substrate.** (a, b, c) Adsorption sites and corresponding adsorption energy on Li (001) (a), LiF (111) (b) and $Li_3Bi$ (111) (c) facets.

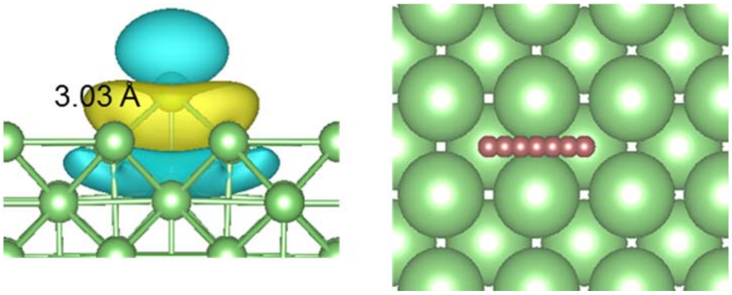


**Supplementary Figure 11 Charge density difference plots showing the adsorption of a Li adatom on the Li (001) facet and the diffusion pathways of Li adatom.**

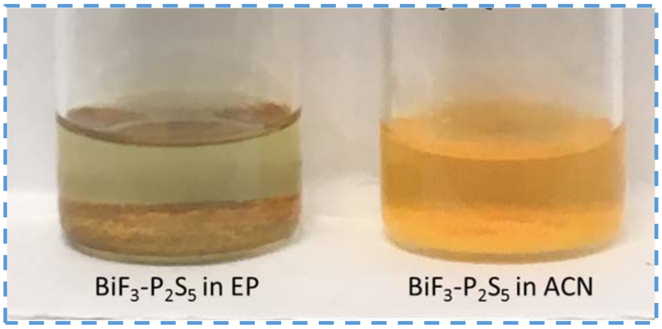


**Supplementary Figure 12 Solvation of $BiF_3$-$P_2S_5$ using different solvents.** Photography of the mixtures of $BiF_3$-$P_2S_5$ (50 mM $BiF_3$) with 1:1 molar ratios in ethyl propionate (left) and acetonitrile (right).

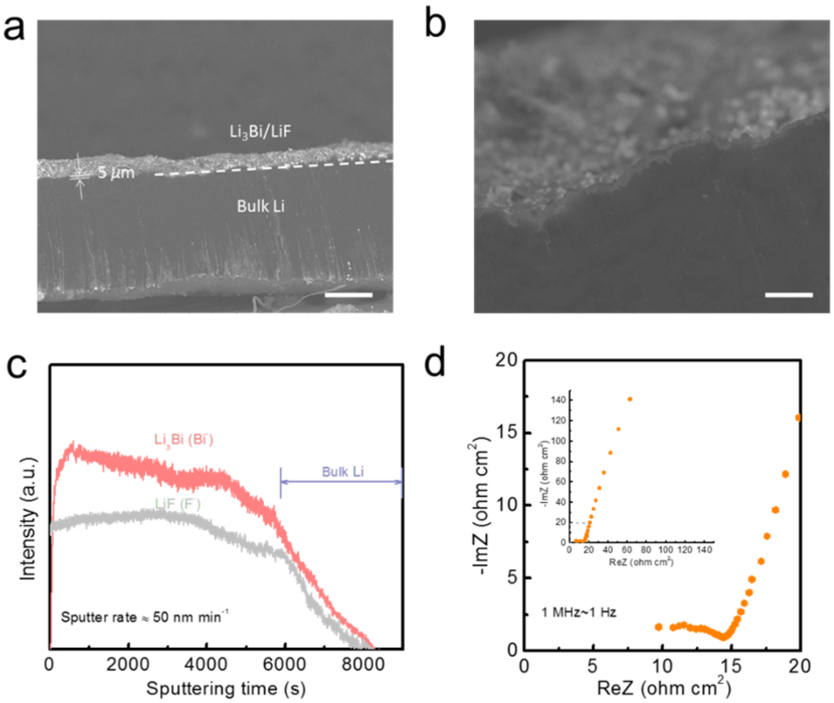


**Supplementary Figure 13 Characterizations of $Li_3Bi/LiF$ layer.** (a, b) SEM images of the cross section of the MFC-Li electrode with different magnifications. (c) TOF-SIMS profile of the MFC-Li surface. (d) EIS profiles of the symmetric cell with the MFC-Li electrodes (without liquid electrolyte and separator). Scale bar for a, b is 200 and 20 µm, respectively.

**Supplementary Note 3**

For the measurement, two MFC-Li electrodes are stacked in CR-2032 coin cell, with their Li-rich sides attached on the stainless steels and their $Li_3Bi/LiF$ layers in direct contact. No liquid electrolyte was added for wetting the electrode. The resulting EIS profile shows semicircle at medium frequencies associated with the total resistance from the bulk, grain boundary and interfaces of the $Li_3Bi/LiF$ composite, and a connected low-frequency tail corresponding to the capacitive behavior. In line with the previous work, we estimate the total Li-ion conduction resistance from the low frequency intercept with the ReZ axis in the EIS profile[2].

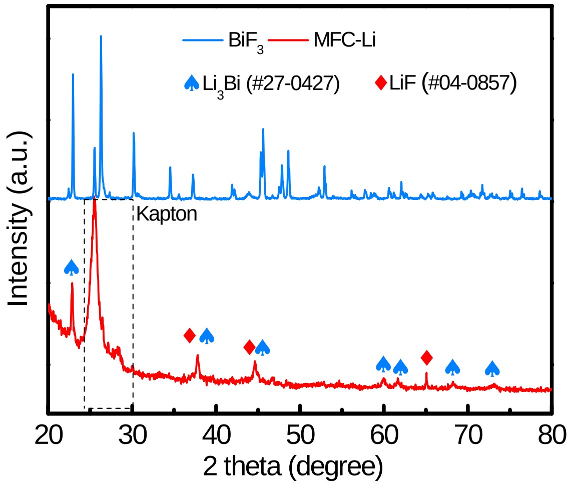


**Supplementary Figure 14 XRD patterns for $BiF_3$ precursor and MFC-Li electrode.**

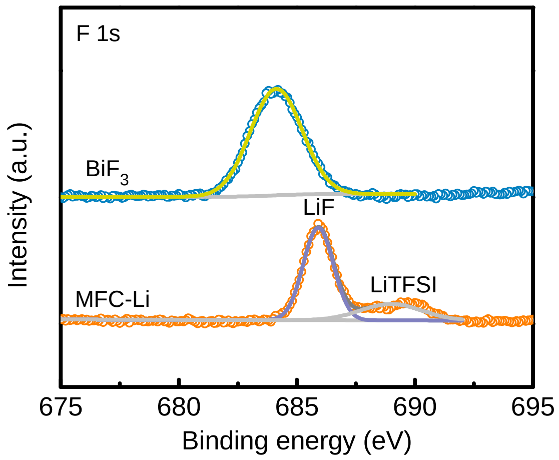


**Supplementary Figure 15 XPS spectra of F 1s for $BiF_3$ precursor and MFC-Li electrode (after sputtering 1800 s).**

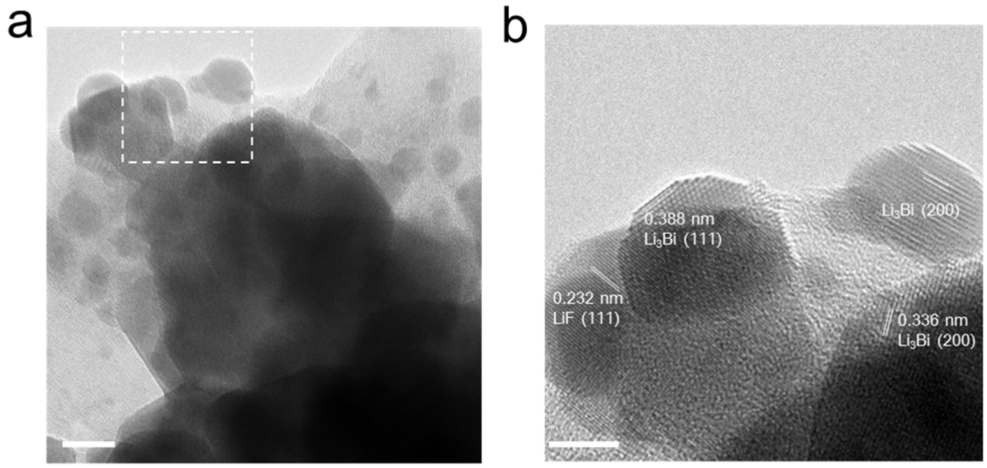


**Supplementary Figure 16 Nanoscale morphology of $Li_3Bi$/LiF composite.** (a) HR-TEM image of the $Li_3Bi$/LiF-based artificial SEI; (b) its enlarged image (the square area in a) with lattices indexed. Scale bar for a and b is 10 nm and 5 nm, respectively.

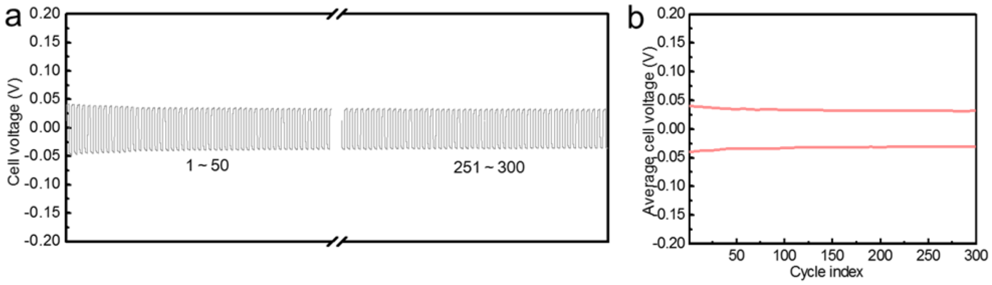


**Supplementary Figure 17 Performance of MFC-Li electrodes in the symmetric cell at 1.0 mA cm$^{-2}$.** (a) Representative cycling voltage profiles at 1.0 mAh cm$^{-2}$ at 1.0 mA cm$^{-2}$ for symmetric cell assembled with the MFC-Li electrode; (b) the average cell voltage versus cycle index.

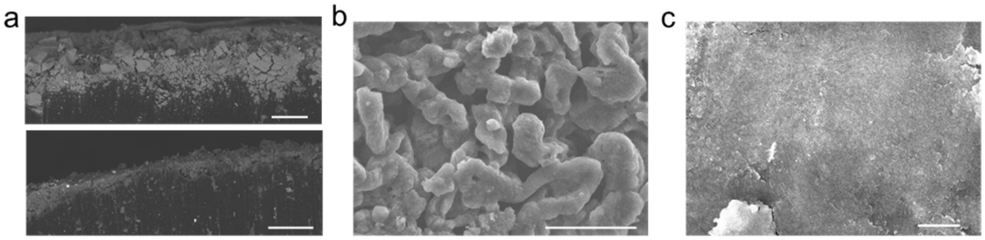


**Supplementary Figure 18 Li plating morphology for different planar anodes.** (a) Cross-sectional view of the cycled Li anode: bare Li metal (79 cycles, up), MFC-Li (300 cycles, down); (b) surface of the cycled bare Li metal, (c) surface of the cycled MFC-Li. The cycling data of bare Li metal can be found in Fig. 5d. Scale bar for a is 100 μm (up) and 50 μm (down); scale bar for b and c is 50 μm and 10 μm, respectively.

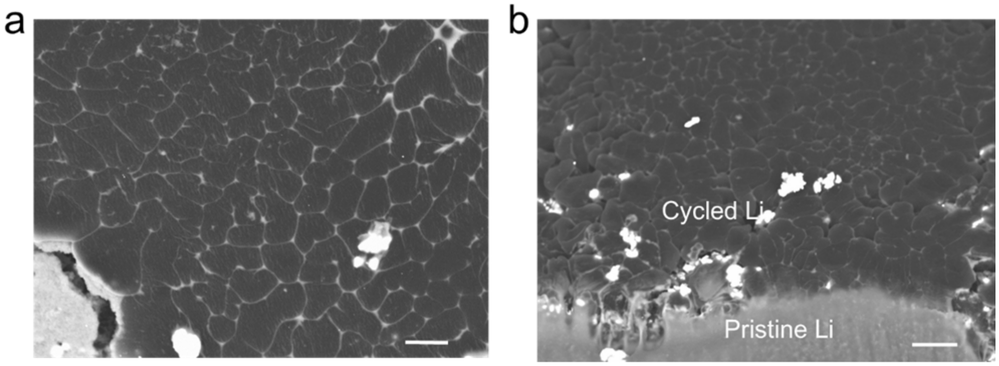


**Supplementary Figure 19 Li plating morphology beneath the $Li_3Bi$/LiF layer.** (a, b) SEM images of the cycled MFC-Li (300 cycles, 1.0 mA cm$^{-2}$, 1.0 mAh cm$^{-2}$, the surface protective layer was stripped by sonification): (a) surface, (b) cross section. Scale bar for a and b is 5 μm and 10 μm, respectively.

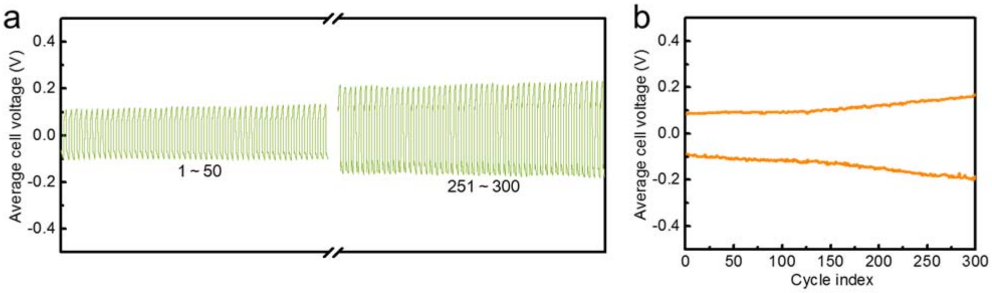


**Supplementary Figure 20 Performance of MFC-Li electrodes in the symmetric cell at 2.5 mA cm$^{-2}$.** (a) Representative cycling voltage profiles at 1.0 mAh cm$^{-2}$ at 2.5 mA cm$^{-2}$ for symmetric cell assembled with the MFC-Li electrode; (b) the average cell voltage versus cycle index. The cell exhibited an almost two-fold increase in overpotential after 300 cycles, showing that the planar MFC-Li electrode is not suitable for being cycled at a higher rate.

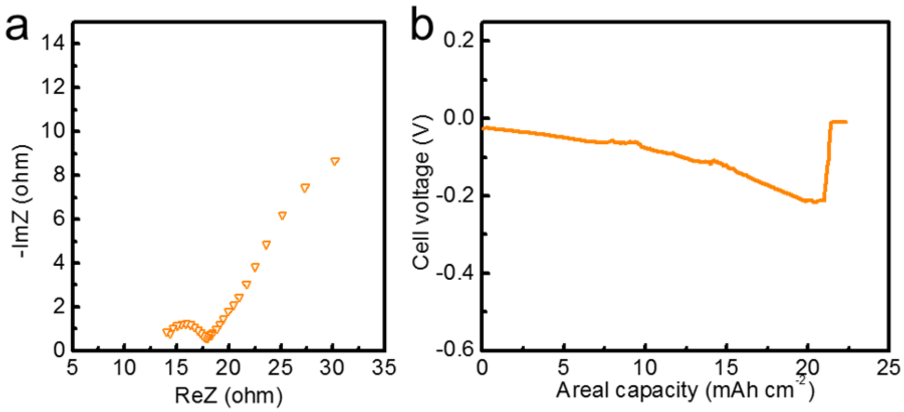


**Supplementary Figure 21 Performance of Li-S/CNF electrode.** (a) EIS measurement results of symmetric cell assembled with the Li-S/CNF electrode (240 μm); (b) long-term discharging profiles before short circuit (0.5 mA $cm^{-2}$) for the symmetric cell with Li-S/CNF electrode.

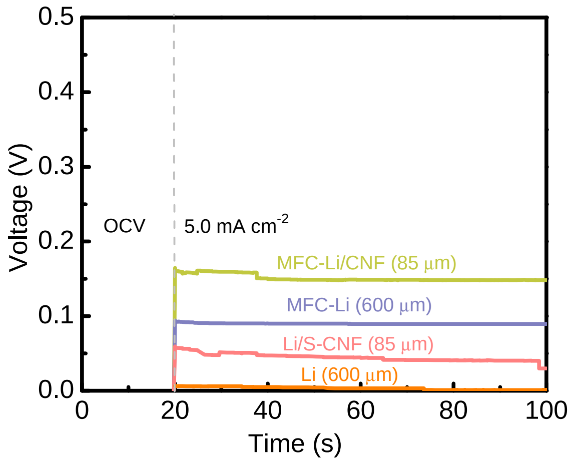


**Supplementary Figure 22 Symmetric cells' polarization curves.** For testing MFC-Li/CNF, MFC-Li and Li/S-CNF and Li, two electrodes are stacked, with their Li-rich sides attached on the stainless steels. No liquid electrolyte was added for wetting the electrode.

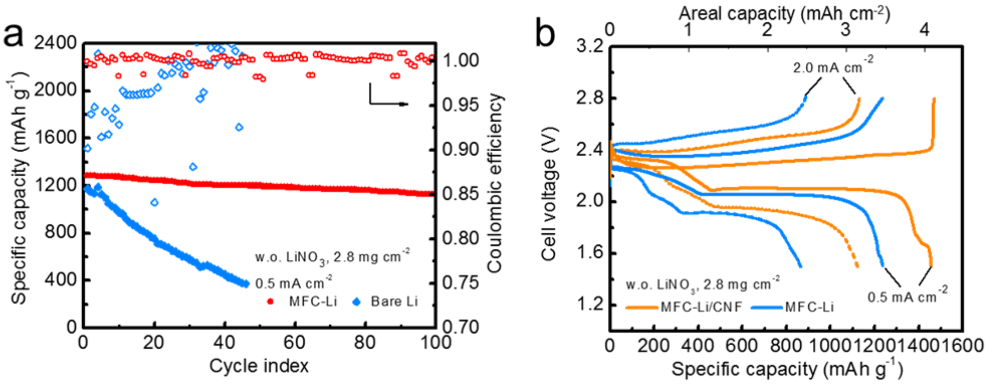


**Supplementary Figure 23 Performance comparison between different anodes.** (a) Cycling performance of Li-S batteries assembled with MFC-Li or bare Li anode at 0.5 mA cm$^{-2}$ (based on the area of cathode) with an areal sulfur loading of 2.8 mg cm$^{-2}$, without the addition of $LiNO_3$. (b) Voltage profiles for the batteries with MFC-Li or MFC-Li/CNF anode. The result shows that there was an effective decrease of overpotential led by the use of porous MFC-Li/CNF electrode.

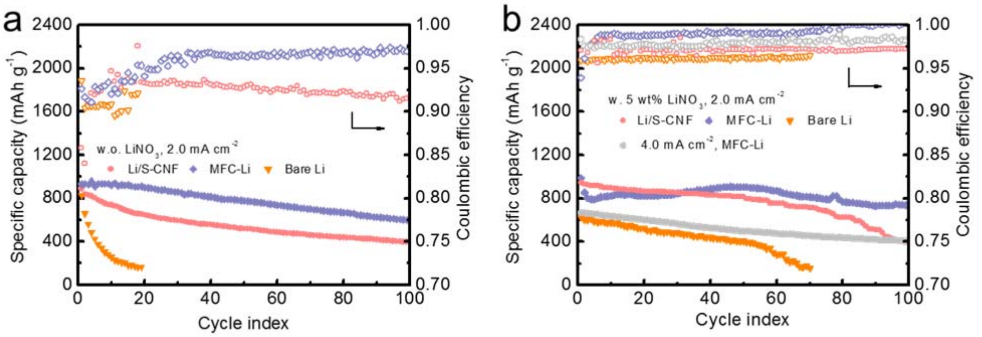


**Supplementary Figure 24 Performance comparison of different anodes with or without $LiNO_3$ additive.** (a) Cycling performance of Li-S batteries assembled with Li-S/CNF, MFC-Li or bare Li anode at 2.0 mA $cm^{-2}$ (based on the area of cathode) with an areal sulfur loading of 2.8 mg $cm^{-2}$, without the addition of $LiNO_3$. (b) Cycling performance achieved with $LiNO_3$ additive (5 wt% $LiNO_3$ in 100 μL liquid electrolyte) at 2.0 or 4.0 mA $cm^{-2}$.

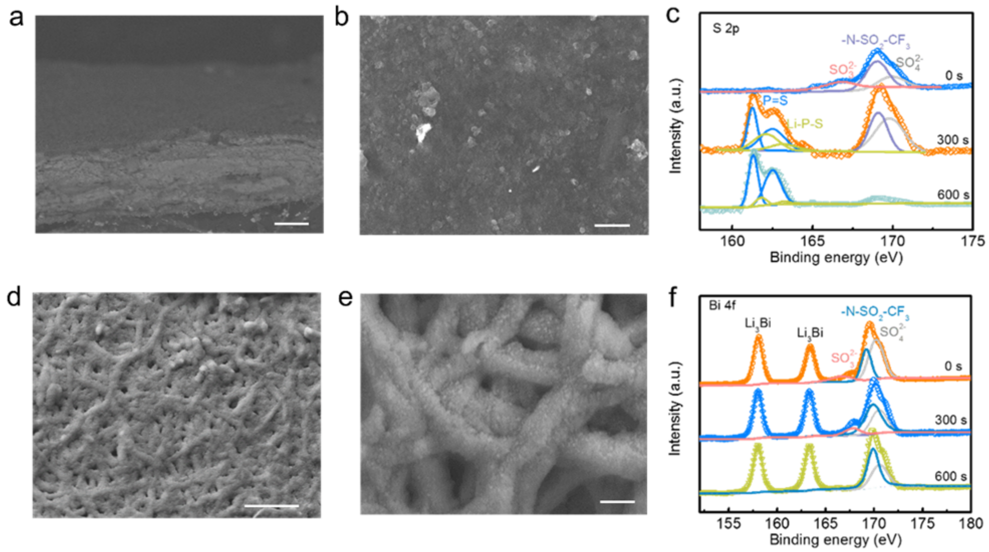


**Supplementary Figure 25 Post-cycling analysis of MFC-Li/CNF.** (a, b) Cross-sectional SEM (a) and top surface SEM (b) of the MFC-Li/CNF after 200 cycles (4.0 mA $cm^{-2}$) in the Li-S battery (6.8 mg $cm^{-2}$ sulfur loading). (c) XPS depth profiling (S 2p) for the top surfaces of MFC-Li/CNF. (d, e) Bottom surface SEM of the MFC-Li/CNF after cycling. (f) XPS depth profiling (Bi 4f) for the bottom surfaces of MFC-Li/CNF. The ranges of S 2p and Bi 4f spectra are overlapped. The Ar sputter speed was 13 nm $min^{-1}$. Scale bar for a, b, d, e is 50 μm,10 μm,10 μm and 1μm, respectively.

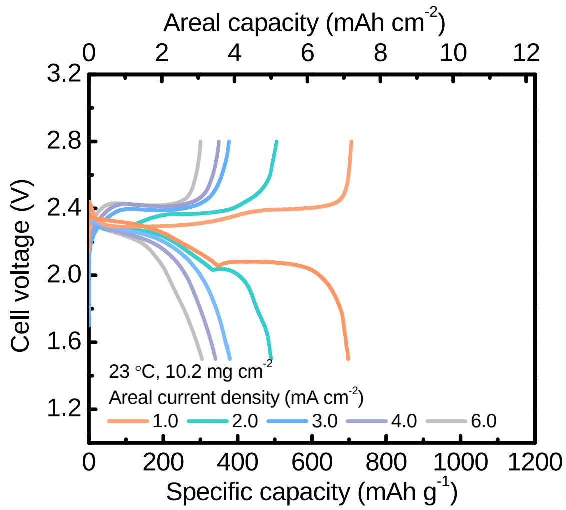


**Supplementary Figure 26 Performance of the high-loading Li-S battery.** Voltage profiles of Li-S batteries assembled with the MFC-Li/CNF electrode and the sulfur/carbon cloth electrode with a sulfur loading of 10.2 mg cm$^{-2}$ (theoretical areal capacity 17.1 mAh cm$^{-2}$) at 23 °C.

**Table R1** A performance comparison between our Li-S battery and those using $LiNO_3$ as electrolyte additives.

| Ref. | Substrate | Surface protection | $LiNO_3$ conc. | Capacity/current | Utilization | Cycle life | Retention |
|---|---|---|---|---|---|---|---|
| Nanda et al.[3] | Cu | N/A | 0.2 M | 0.84 mAh $cm^{-2}$ (0.84 mA $cm^{-2}$) | 17.9% | 100 | 51.5% |
| Liu et al. [4] | Cu | DND-polymer | 0.3 M | 0.90 mAh $cm^{-2}$, (1.25 mA $cm^{-2}$) | 18% | 400 | 67% |
| Chen et al.[5] | Li foil | Alucone | 0.3 M | 3.5 mAh $cm^{-2}$, (0.95 mA $cm^{-2}$) | 2.9% | 140 | 94% |
| Cha et al. [6] | Li foil | $MoS_2$ | 0.25 M | 3.8 mAh $cm^{-2}$, (2.9 mA $cm^{-2}$) | 15.4% | 1200 | 85% |
| Tang et al.[7] | Li foil | $Li_xSi$ | 0.1 M | 1.82 mAh $cm^{-2}$, (1.675 mA $cm^{-2}$) | 1.5% | 160 | 91% |
| Liu et al.[8] | Co/N-PCNS | N/A | 0.15 M | 1.2 mAh $cm^{-2}$, (0.33 mA $cm^{-2}$) | 40% | 60 | 68% |
| Cai et al.[9] | TCF | Doped carbon shell | 0.3 M | 4.0 mAh $cm^{-2}$, (2.73 mA $cm^{-2}$) | 30% | 200 | 77.5% |
| Chang et al.[10] | Carbon cloth | Nano Cu | 0.3 M | 2.4 mAh $cm^{-2}$, (2.0 mA $cm^{-2}$) | 40% | 250 | 72.5% |
| This work | CNF | $Li_2S$-$P_2S_5$ solid electrolyte | N/A | 5.4 mAh $cm^{-2}$, (0.85 mA $cm^{-2}$) | 41% | 200 | 90.7% |
| | | $Li_3Bi$/LiF artificial layer | | 6.4 mAh $cm^{-2}$, (3.40 mA $cm^{-2}$) | 48.4% | 200 | 91.5%* |
| | | | | 6.0 mAh $cm^{-2}$, (5.1 mA $cm^{-2}$) | 45.4% | 200 | 91.0%* |

*Abbreviated items:*

*DND: double-layer nanodiamond; Co/N-PCNS: cobalt-embedded nitrogen-doped porous carbon nanosheets; TCF: tubular carbon fabric.*

** The capacity exhibited slight increase in the initial tens of cycles (Fig. 6e). Therefore, the capacity retention is calculated based on the ratio of capacity at the last cycle and the maximum capacity over cycling.*

**Supplementary Note 4**

We compare our work to representative literatures (Supplementary Table 1), with an emphasis on their effectiveness in achieving stable cycling in a Li-S battery. We reveal that the three essential requirements (porous scaffold, surface protective layer and artificial SEI) for stabilizing Li anode were met simultaneously through our designed spontaneous reactions. The roles of each composition (LiF, $Li_3Bi$ and $Li_2S$-$P_2S_5$ based solid electrolyte) were well defined, which proofed to alleviate the issues of dendrite and parasitic reactions. Owing to the rational electrode structures and compositions, for the as-developed Li-S full batteries, the Li anode could be cyclable with a high Li utilization and a low capacity fading rate, among the highest performance reported to-date.

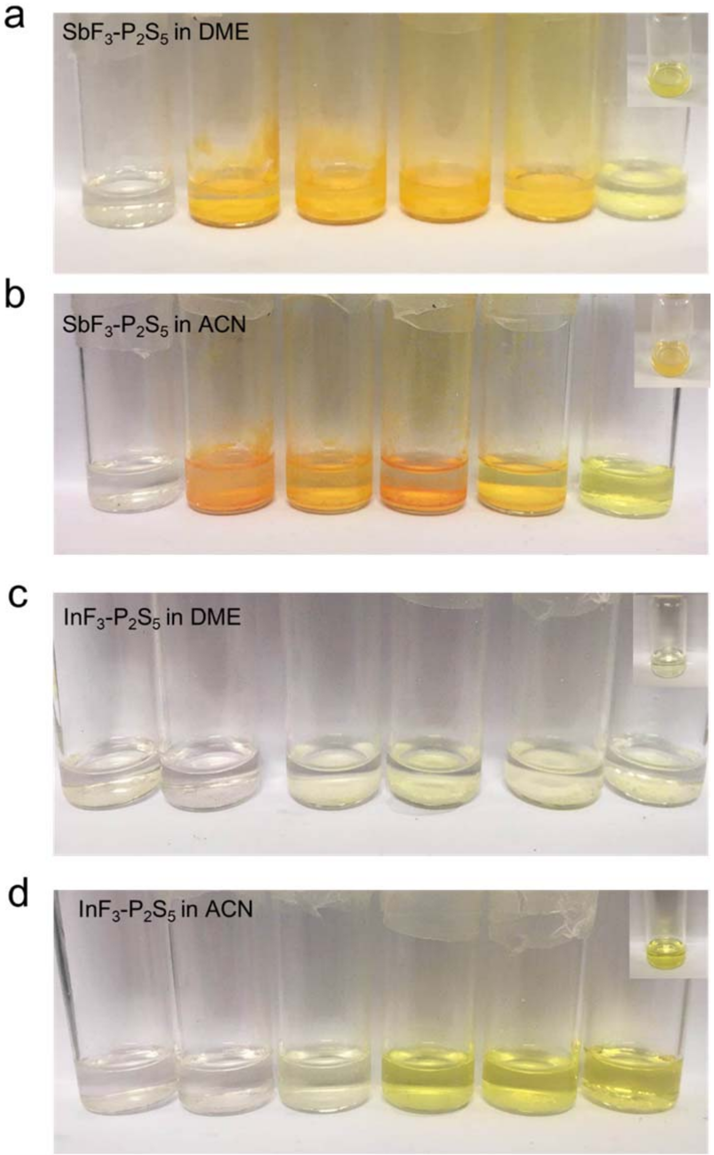


**Supplementary Figure 27 Solvation of $SbF_3$ and $InF_3$ using $P_2S_5$.** Photography of the mixtures of $SbF_3$-$P_2S_5$ (a, b) and $InF_3$-$P_2S_5$ (c, d) (50 mM metal fluoride) with different molar ratios (1:0, 1:0.25, 1:0.5, 1:1, 1:2, 0:1, from left to right) in DME and ACN respectively. The inset figures show the supernant solution with a metal fluoride: $P_2S_5$ molar ratio of 1:1.

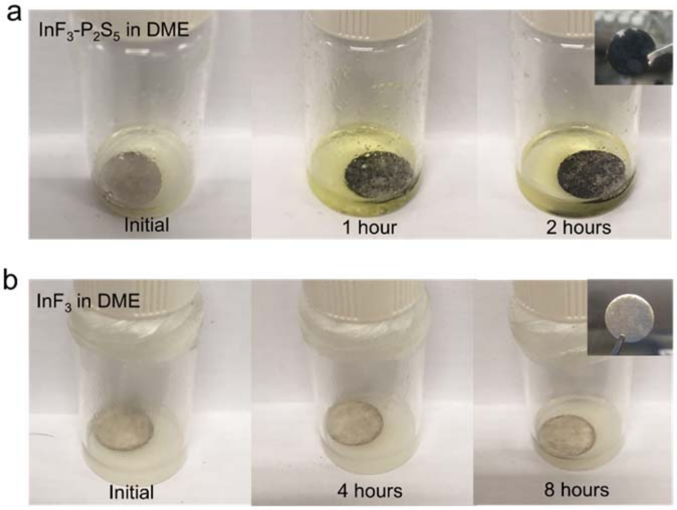


**Supplementary Figure 28 Reaction between solvated $InF_3$ and Li.** (a, b) Photography showing the spontaneous reaction between Li and $InF_3$ (50 mM) solvated with $P_2S_5$ in DME (a); and the control group with $InF_3$ only in DME (b). The inset photos show the Li foils after soaking. The surface passivation of Li can complete after 2-hour soaking using $InF_3$-$P_2S_5$ (1:1) at 45 °C.

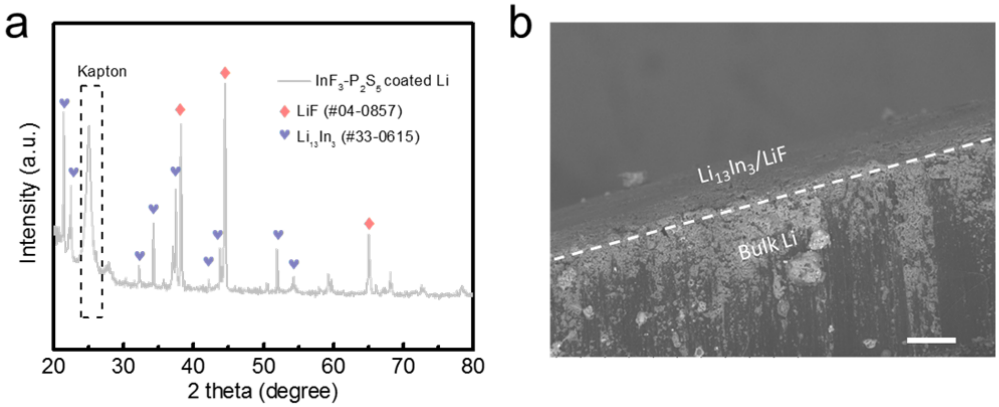


**Supplementary Figure 29 Characterizations of Li metal coated with $Li_{13}In_3$ and LiF.** (a) XRD patterns for the as-prepared $InF_3$-$P_2S_5$ coated Li electrode; (b) SEM image of the cross section of the $InF_3$-$P_2S_5$ coated Li electrode. Scale bar for b is 100 μm.

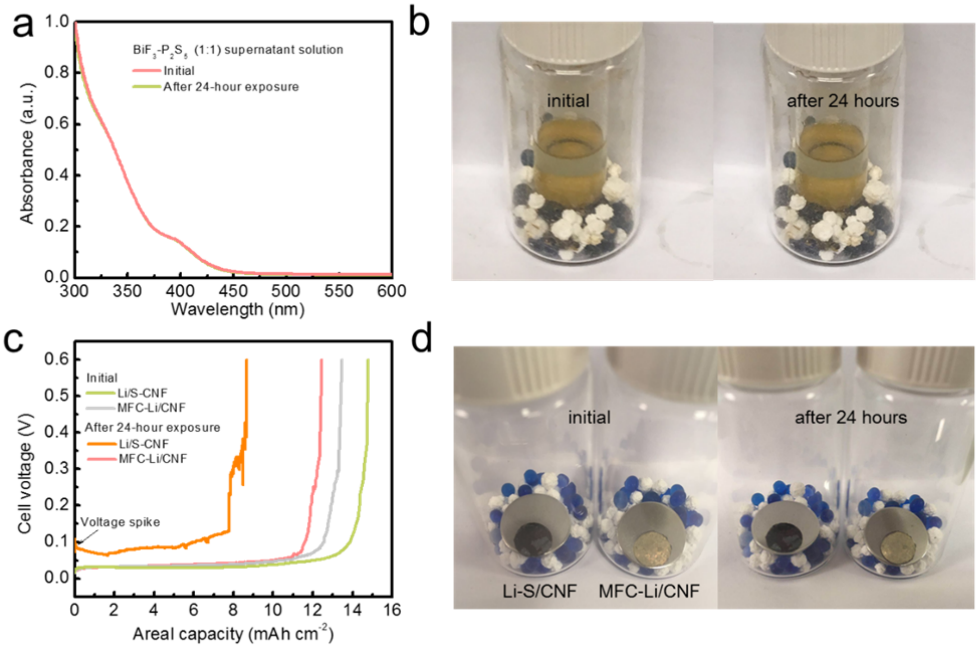


**Supplementary Figure 30 Dry air stability test.** (a) UV-Vis spectra of the supernatant solution of $BiF_3$-$P_2S_5$ before and after dry air exposure (diluted to 25% of its initial concentration). (b) Photography of the $BiF_3$-$P_2S_5$ in DME (50 mM $BiF_3$) in the dry air environment. (c) Li stripping curves of Li-S/CNF and MFC-Li/CNF before and after dry air exposure. (d) Photography of the electrodes in the dry air environment.

**Supplementary Note 5**

Anhydrous $CaCl_2$ (200 mg) was put in the vessel (20 mL) to create dry air environment. The electrodes (8 mm in diameter) for testing were punched from the same Li/S-CNF or MFC-Li/CNF pellet (18 mm), to guarantee that their properties were almost identical. The nitrogen and oxygen contained in the vessel are excessive, which can fully react ~34 mg Li to form $Li_3N$ and $Li_2O$.

## References


1. Jain, A. *et al.* Commentary: The Materials Project: A materials genome approach to accelerating materials innovation. *Apl Mater.* **1,** 11002–11011 (2013).

2. Fu, K. K. *et al.* Toward garnet electrolyte–based Li metal batteries: An ultrathin, highly effective, artificial solid-state electrolyte/metallic Li interface. *Sci. Adv.* **3,** (2017).

3. Nanda, S., Gupta, A. &Manthiram, A. A Lithium–Sulfur Cell Based on Reversible Lithium Deposition from a Li2S Cathode Host onto a Hostless-Anode Substrate. *Adv. Energy Mater.* **8,** 1801556 (2018).

4. Liu, Y. *et al.* An Ultrastrong Double-Layer Nanodiamond Interface for Stable Lithium Metal Anodes. *Joule* **2,** 1595–1609 (2018).

5. Chen, L. *et al.* Directly formed alucone on lithium metal for high-performance Li batteries and Li–S batteries with high sulfur mass loading. *ACS Appl. Mater. Interfaces* **10,** 7043–7051 (2018).

6. Cha, E. *et al.* 2D MoS 2 as an efficient protective layer for lithium metal anodes in high-performance Li–S batteries. *Nat. Nanotechnol.* **13,** 337–344 (2018).

7. Tang, W. *et al.* Lithium Silicide Surface Enrichment: A Solution to Lithium Metal Battery. *Adv. Mater.* **30,** 1801745 (2018).

8. Liu, S. *et al.* Superhierarchical Cobalt-Embedded Nitrogen-Doped Porous Carbon Nanosheets as Two-in-One Hosts for High-Performance Lithium–Sulfur Batteries. *Adv. Mater.* **30,** 1706895 (2018).

9. Cai, W. *et al.* The Dual-Play of 3D Conductive Scaffold Embedded with Co, N Codoped Hollow Polyhedra toward High-Performance Li–S Full Cell. *Adv.*

*Energy Mater.* **8,** 1802561 (2018).

10. Chang, J. *et al.* Flexible and stable high-energy lithium-sulfur full batteries with only 100% oversized lithium. *Nat. Commun.* **9,** 4480 (2018).
